# Safety and Tolerability of Ivermectin and Albendazole Mass Drug Administration in Lymphatic Filariasis Endemic Communities of Tanzania: A Cohort Event Monitoring Study

**DOI:** 10.3390/ph15050594

**Published:** 2022-05-12

**Authors:** Adam M. Fimbo, Omary Mashiku Minzi, Bruno P. Mmbando, Parthasarathi Gurumurthy, Appolinary A. R. Kamuhabwa, Eleni Aklillu

**Affiliations:** 1 Division of Clinical Pharmacology, Department of Laboratory Medicine, Karolinska Institutet, Karolinska University Hospital, 141 86 Huddinge, Stockholm, Sweden; adamfimbo@gmail.com; 2 Tanzania Medicines and Medical Devices Authority (TMDA), Dar es Salaam P.O. Box 77150, Tanzania; 3 Department of Clinical Pharmacy and Pharmacology, School of Pharmacy, Muhimbili University of Health and Allied Sciences, Dar es Salaam P.O. Box 65013, Tanzania; minziobejayesu@gmail.com (O.M.M.); enali2012@gmail.com (A.A.R.K.); 4 National Institute for Medical Research, Tanga Center, Tanga P.O. Box 5004, Tanzania; b.mmbando@yahoo.com; 5 Pharmacovigilance and Clinical Trials, Botswana Medicines Regulatory Authority, Gaborone 999106, Botswana; partha18@gmail.com

**Keywords:** cohort event monitoring, active safety surveillance, ivermectin, albendazole, lymphatic filariasis, mass drug administration, pharmacovigilance, Neglected Tropical Diseases, preventive chemotherapy, Tanzania

## Abstract

Ivermectin and albendazole (IA) combination preventive chemotherapy to all at-risk populations is deployed to eliminate lymphatic filariasis. Although safety monitoring is imperative, data from Sub-Saharan Africa is scarce. We conducted a large-scale active safety surveillance of adverse events (AEs) following IA mass drug administration (MDA) to identify the type, incidence, and associated risk factors in Tanzania. After recording sociodemographic, clinical, and medical histories, 9640 eligible residents received single-dose IA combination preventive chemotherapy. Treatment-associated AEs were actively monitored through house-to-house visits on day 1, day 2, and day 7 of MDA. Events reported before and after MDA were cross-checked and verified to identify MDA-associated AEs. 9288 participants (96.3%) completed the seven-day safety follow-up, of whom 442 reported 719 MDA-associated AEs. The incidence of experiencing one or more type of MDA-associated AE was 4.8% (95% CI = 4.3–5.2%); this being significantly higher among those with Pre-MDA clinical events than those without (8.5% versus 4.1%, *p* < 0.001). AEs were mild (83.8%), moderate (15.9%), and severe (0.3%), and most resolved within 72 h. The incidence of experiencing one, two, ≥ three types of AEs were 2.8%, 1.3%, and 0.6%, respectively. The most common AEs were headache (1.23%), drowsiness (1.15%), fever (1.12%), and dizziness (1.06%). A chronic illness, or clinical manifestation of lymphatic filariasis, or being female or pre-existing clinical symptoms were independent significant predictors of AEs. IA combination preventive chemotherapy is safe and tolerable, and associated AEs are mild-to-moderate and transient, with few severe AEs. Safety monitoring during MDA campaigns in individuals with underlying clinical conditions is recommended for timely detection and management of AEs.

## 1. Introduction

Lymphatic filariasis (LF) is a painful and disfiguring disease caused by three species of thread-like nematode worms, namely Wuchereria bancrofti, Brugia malayi and Brugia timori. Wuchereria bancrofti is the most common etiological agent and accounts for over 90% of infections in Sub Saharan Africa (SSA) [1,2]. LF infection can be acquired during childhood, but the visible manifestations of LF may only occur later in life [3]. Clinical manifestations include lymphoedema of the arms or legs and genital diseases (hydrocele, chylocele, and swelling of the scrotum and penis) [2,4].

The World Health Organization (WHO) recommends periodic Mass Drug Administration (MDA) of anti-helminthics to all at-risk populations in endemic countries as preventive chemotherapy to halt the transmission of infection and eliminate LF [1,5]. In 2020, 862.3 million people required preventive chemotherapy for the elimination of LF globally [6]. Ivermectin and albendazole (IA) combination therapy is deployed in MDA campaigns for elimination of LF. Combinations consisting of diethylcarbamazine (DEC) are also available but are not recommended in areas where onchocerciasis or loiasis is co-endemic with LF. Single-dose Ivermectin therapy is effective in clearing microfilariae (mf) from circulation [7]. During MDA campaigns, drugs are distributed to communities on a large scale without a prior diagnosis of diseases and safety follow-up. All at-risk populations receive MDA, and distribution is generally random to consented individuals. Safety monitoring during MDA is recommended, but the NTD programs are mainly focusing on drug coverage and the decline of infection rates, and pharmacovigilance during MDA campaigns is almost nonexistent [8].

The Neglected Tropical Diseases Control Program (NTDCP) in Tanzania has been coordinating and implementing the MDA of IA combination preventive chemotherapy since 2002 [9]. In this respect, 16 MDA rounds had been completed by the time this study was conducted. However, as distribution is usually random, whether the same individuals are always exposed on each round remains to be cross-examined. National medicines regulatory authorities in Africa, including the Tanzania Medicines and Medical Devices Authority (TMDA) strive to obtain safety data after marketing authorization of many drugs [10,11]. A recent comparative assessment of the pharmacovigilance systems in the NTD Programs in four East African countries indicated that no adverse events following MDA were reported to the National Medicines Regulatory Authorities despite millions of people being exposed to MDA in the year 2017/2018 [12]. Various initiatives have been undertaken to reverse the status-quo, and different methods have been introduced to increase the number of reports. The WHO currently recommends the adoption of cohort event monitoring (CEM) as the preferred pharmacovigilance method for safety surveillance of medicines, particularly in areas where passive or voluntary reporting is challenging [13,14]. The WHO recommends safety monitoring in public health interventions using CEM at baseline and seven days post-treatment [15].

Large-scale safety surveillance studies are pivotal in detecting rare severe adverse events [16]. Although millions of people in Africa are receiving periodic MDA, safety data from large-scale active CEM studies are scarce partly due to the lack of fully functional pharmacovigilance systems in the region [10,11,12]. The WHO’s global individual case safety reports database (VigiBase) recently reported serious suspected ADRs and safety signals associated with ivermectin use and the under-reporting of ADRs in SSA [17]. Under or incomplete reporting of AEs, lack of information on baseline characteristics embracing the number of drug administrations, and the absence of denominators are major drawbacks inherent in spontaneous reporting pharmacovigilance databases such as VigiBase [18]. The CEM in drug safety assessment studies overcomes such drawbacks.

The safety profile of drugs used in public health programs vary between individuals and populations due to host-genetic and environmental factors, including coinfection, comorbidity, and drug-interactions and the use of traditional medicines, which is common in Africa [19,20,21,22,23]. Poor safety surveillance of medicines during MDA campaigns and the under-reporting rate of AEs in SSA makes it challenging to accurately estimate the risks of drugs used in MDA to inform healthcare policy and practice. Therefore, we conducted a large-scale active CEM study to identify the incidence, type and risk factors of AEs following IA combination preventive chemotherapy during the MDA campaign in Tanzania.

## 2. Results

### 2.1. Baseline Characteristics of the Study Population

A total of 9640 eligible participants from 24 villages who consented to participate in the study were enrolled. Sociodemographic and medical histories, including comorbidities, active and chronic infections, clinical symptoms, comedications, use of traditional and herbal medicines, etc. were recorded before taking MDA (pre-MDA events). Study participants received IA combination preventive chemotherapy through MDA campaigns coordinated and led by the NTDCP. Out of the 9640 enrolled participants, 9288 (96.3%) completed the seven-day safety follow-up. Among 9288 individuals from whom post-MDA safety data were collected, 4816 (51.9%) were females. Sociodemographic and baseline characteristics of study participants are presented in Table 1.

### 2.2. Incidence of Adverse Events Following Ivermectin and Albendazole MDA

**Figure 1:**
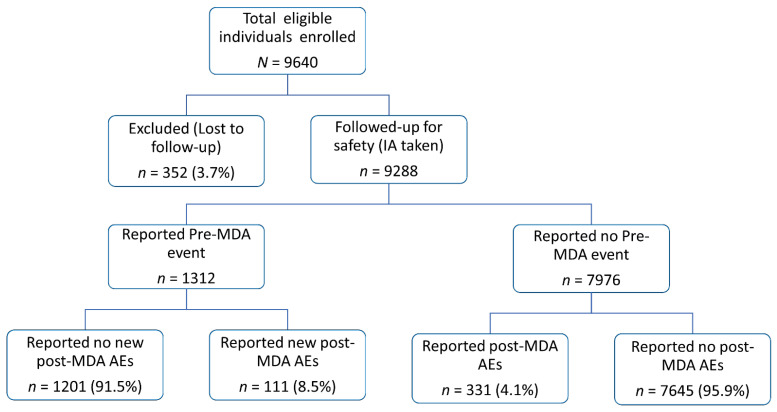
Study flow chart of participant enrolment and follow-up.

A total of 9288 participants who received IA during MDA completed the safety follow-up and were included in the analysis. A total of 352 (3.7%) individuals were lost to follow-up and were not included in the analysis. A baseline assessment of self-reported symptoms through interviews was recorded before drug administration, and 1312 individuals reported clinical symptoms before taking MDA (pre-MDA event). Any reported post MDA event was crosschecked and verified to differentiate MDA-associated AEs from pre-existing clinical symptoms. The study flow chart and proportion of participants who reported pre- and post-MDA events is presented in Figure 1. Out of the 9288 from which follow-up data were recorded, 442 individuals reported at least one post-MDA AE. The overall incidence of experiencing at least one post-MDA AE was 4.8% (95% CI = 4.3–5.2%). The proportion of individuals who reported one, two and three or more events were 2.8% (*n* = 260), 1.3% (*n* = 122), and 0.6% (*n* = 60), respectively.

Of the 1312 individuals who reported any clinical symptom before taking MDA, 111 (8.5%, 95% CI = 4.3–5.2%) reported at least one new symptom after taking MDA (post-MDA AE). Among 7976 individuals who did not report pre-MDA symptoms, 331 (4.1%, 95% CI = 3.7–4.6%) experienced at least one type of post-MDA AE. Participants with underlying pre-MDA clinical symptoms had a significantly higher risk of experiencing MDA-associated AE (*p* < 0.001, odds ratio = 2.13: 95% CI = 1.71–2.67). The incidence of AEs decreased by day during the follow-up period compared to day one (*p* < 0.001).

### 2.3. Inicdence Strafied by Types of MDA-Associated Adverse Events

**Figure 2:**
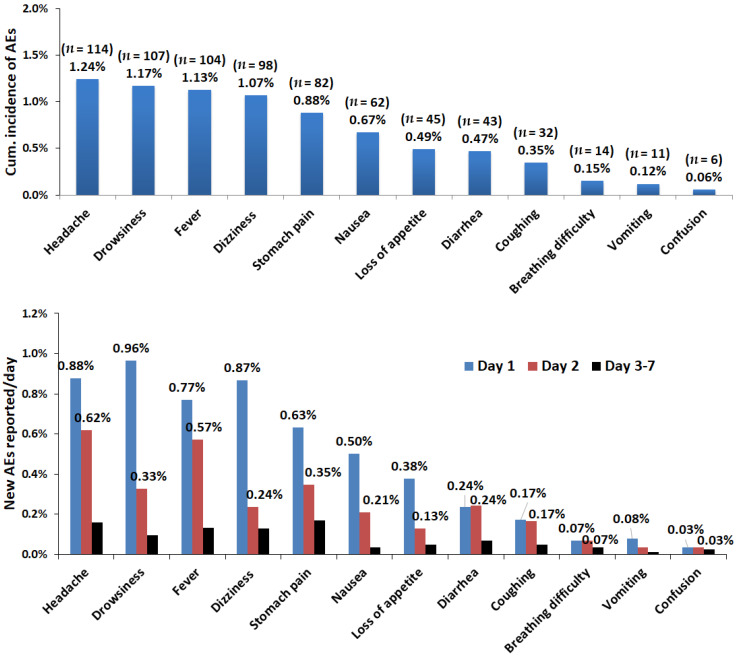
Cumulative incidence of each type of post-MDA AEs over seven days (top) and incidence of new AEs reported per day (bottom) following MDA.

Generally, more AEs were observed during the first 24 h of MDA (day one) and decreased progressively until day seven. The most common AEs with relatively higher incidence rates were headache (1.23%), drowsiness (1.15%), fever (1.12%), dizziness (1.06%), and abdominal pain (0.88%), while confusion, vomiting, and difficulty in breathing had the lowest incidence rates throughout the follow-up period. The cumulative incidence of each type of reported post-MDA during the seven-day follow-up period and stratified by date of occurrence after MDA is presented in Figure 2.

### 2.4. Severity Grading of MDA-Associated Adverse Events

Most of the reported post-MDA AEs were mild (83.8%) and moderate (15.9%), with few that were severe (0.3%). Only two individuals reported severe drowsiness (0.9%) and dizziness (1%). Table 2 summarizes the severity grading of each type of reported AE following MDA.

### 2.5. Correlates and Predictors of Adverse Events Following MDA

The incidence rates of AEs were significantly higher among females and those who had a chronic illness and the chronic manifestation of LF (*p* < 0.001). The incidence of AEs was not statistically different between the different age groups, those who used bed nets and those who participated in the previous MDA round. Likewise, AEs was not significant associated with the use of traditional medicines or the number of Ivermectin tablets administered. Table 3 provides the chi-square test statistics of the incidence and correlates of AEs following MDA.

Risk factors associated with the development of one or more AE following MDA were further explored in a univariate and multivariate regression analysis. Using binomial regression analysis, being female (*p* < 0.001), having a chronic illness (*p* = 0.001), and chronic manifestation of LF (*p* = 0.005) were significant predictors of AEs following MDA. Results from the univariate and multivariate regression analysis is presented in Table 4.

### 2.6. Chronic Clinical Conditions and Their Association with Adverse Events

Chronic conditions and their association with reported AEs were further assessed in a multivariate logistic regression analysis. Frequency and odds ratios of AEs in individuals with chronic conditions are presented in Table 5. Having hypertension and asthma were significantly associated with higher incidences of AEs. Chronic kidney problems, diabetes, and tuberculosis were not associated with AEs. Fever, dizziness, stomach pain, diarrhea, breathing difficulty, vomiting and confusion were mostly seen in those with hypertension, while headache, dizziness, loss of appetite, difficulty in breathing, and vomiting were more observed in asthmatic patients.

## 3. Discussion

We conducted a large-scale active safety surveillance study to identify the incidence, timing, type, severity, and associated risk factors of AEs following IA combination preventive chemotherapy in LF endemic communities [24]. Any clinical symptoms before drug intake were recorded and cross-checked with post-MDA reported events in each study participant to differentiate treatment-associated AEs from any pre-existing clinical symptoms. The overall cumulative incidence of experiencing at least one type of MDA-associated AE was 4.8%; this being significantly higher among those who had pre-existing clinical conditions (8.5%) than those without (4.1%). The incidence of experiencing one, two, and three or more types of AEs were 2.8%, 1.3%, and 0.6%, respectively. Most of the observed AEs were mild (83.8%) and moderate (15.9%), with few severe (0.3%). Pre-existing clinical symptoms, chronic manifestations of LF, chronic illness, and female sex were significant risk factors associated with AEs following MDA of IA preventive chemotherapy.

Most AEs that occurred during the first two days of MDA were transient and resolved progressively through day seven. Headache, drowsiness, fever, dizziness, and stomach pain were the most common AEs (Figure 2). Previous clinical trials and observational studies reported similar common AEs, including headache, pruritus, muscle pain, coughing, dyspnea, nausea, vomiting, diarrhea, confusion, and skin reactions [7]. The same events were reported in higher numbers through spontaneous reporting to the WHO’s Vigibase, although the incidence rate could not be quantified [17]. Safety studies conducted elsewhere using diverse study designs, target populations, and treatment indications reported varying incidence rates of mild-to-moderate AEs in the first two days of treatment [25,26,27,28]. A higher incidence (>15%) of mild-to-moderate AEs following MDA with diethylcarbamazine containing triple therapy (diethylcarbamazine + ivermectin + albendazole) or dual therapy (diethylcarbamazine + albendazole) is reported [19,29,30,31]. The relatively lower incidence of AEs in our study indicates a better safety profile of IA combination chemotherapy than diethylcarbamazine containing dual or triple chemotherapy for the elimination of LF.

Interestingly, we found sex differences in the incidence rates of AEs in our study. The significantly higher incidences of AEs in females than males could be due to both sex and gender-related factors. Previous studies reported that women experience more AEs than males [31,32,33]. Sex differences in pharmacokinetics have been reported to predict ADRs [34]. Women display a two-fold higher risk of developing ADRs and are more likely to be hospitalized secondary to ADRs than men [35]. Depending on the type and severity of reactions, hormonal changes, and pharmacokinetic properties, dose adjustments are recommended for women in clinical practice. However, sex-dependent variation in Ivermectin and albendazole pharmacokinetics and their association with susceptibility to treatment-associated AEs remains to be investigated. Apart from sex-related factors, differences in social behaviors, lifestyle factors, health information-seeking behavior and adherence may also lead to gender-specific differences in perception, occurrence, and reporting of ADRs [36]. Women are keen and tend to follow-up on their health situations and changes more often than men. Underlying gender-related differences in reporting of potential ADRs from the WHO global database (VigiBase) between 1967 and 2018 was reported [36]. The authors noted that women from puberty and onwards and especially those in their reproductive age reported more ADRs than men.

We found that having a chronic manifestation of LF as a significant risk factor for AEs following IA combination chemotherapy. This is in line with previous studies reporting a higher incidence of treatment-associated systemic AEs related to the death of microfilariae in LF-infected individuals than those without the disease [7]. Circulating filarial antigens (CFA) and microfilaremia (mf) positivity and high mf density were significantly associated with higher rates of AEs following MDA with albendazole + diethylcarbamazine + Ivermectin [29]. The increase in cytokine, filarial DNA, and CFA levels were related to the development of AEs following treatment of LF [37]. Other studies have shown a tendency for slightly more AEs in LF patients treated with the combination of Ivermectin and albendazole than for albendazole alone [25]. Thus, safety monitoring in those with a chronic manifestation of LF during MDA is recommended for proper management.

We found no significant association of AEs with age groups, the use of traditional medicines or the number of Ivermectin tablets taken. This finding is somehow different from a study done in Kenya that reported an increased number of diethylcarbamazine tablets (≥3 tablets) was significantly correlated with increased risk for AEs [19]. Nonetheless, our study drugs were the IA combination used during MDA campaigns in Tanzania as compared to the diethylcarbamazine containing regimen used in Kenya. Chronic illness, in particular hypertension and asthma, were significant risk factors of AEs. The frequency of AEs embracing fever, dizziness, stomach pain, diarrhea, breathing difficulty, vomiting, and confusion was mostly seen in hypertensive patients. The association between venous hypertension and lymphoedema was reported previously due to increased capillary filtration and inflammation [38]. The association of asthma with AEs in our study could be due to Tropical Pulmonary Eosinophilia triggered by tripped microfilariae in the lung alveoli and bronchioles [39]. Eosinophilia was also frequently reported with Ivermectin use in the review of Vigibase [17]. Headache, dizziness, loss of appetite, difficulty in breathing, and vomiting were AEs mostly observed in those with asthma. Other risk factors such as kidney disease, diabetes and TB were not associated with AEs. Ivermectin is also eliminated exclusively in faeces, and less than 1% of the drug is excreted unchanged in the urine [40]. Therefore, renal insufficiency may have little impact on pharmacokinetics and the possible toxicity of the drug. Conversely, other studies have reported abnormalities in the kidneys caused by microfilariae and adult worms in LF endemic areas [7,41], which nonetheless was not the case in our study.

Collation and amassing of safety information had been effectively possible through a high follow-up rate. Only 352 out of 9640 individuals (3.7%) were lost to follow-up (Figure 1). Considering the under-reporting observed in passive surveillance approaches, CEM designs using active methods of follow-up proves to be pivotal in pharmacovigilance systems, especially in resource-limited settings. A close collaboration between public health programs and national medicine regulatory authorities is critical to integrating pharmacovigilance in MDA campaigns and practice [8]. Through experiences gained, the regulatory authority and NTD program in Tanzania need to collaborate on safety monitoring during MDA campaigns for timely detection and management of AEs and to promote public health. Through this study we demonstrated the feasibility of active safety surveillance in the MDA program through effective collaboration between academia, national medicine regulators, and public health programs in an African setting.

To our knowledge, this is the first large-scale active cohort event monitoring study to investigate the incidence and associated risk factors of AEs following the MDA of IA in Tanzania and SSA. The strength of our study is the large sample size, thereby detecting rare severe AEs, and the seven-day follow-up period to identify the time curse as recommended by WHO. This has enabled us to quantify the incidence and timing of each type of AE. However, as MDA is given to all eligible individuals living in LF endemic areas without a prior diagnosis, we could not compare the incidence of AEs between mf and CFA positives versus healthy individuals; and this may be considered our study limitations. Nevertheless, we found a significantly higher incidence of AEs among patients with chronic LF manifestations than those without. Our study provides relevant information for national and international stakeholders on the safety of IA preventive chemotherapy to eliminate LF.

## 4. Materials and Methods

### 4.1. Study Setting and Population

This safety study was conducted in Mkinga district, Tanga region, Tanzania. The district was selected for the study because of the high LF endemicity observed since 2002. Most of the population in the district had access to a health facility within 6 km. The main economic activities include fishing, subsistence farming, low-scale livestock keeping, and minor trading for the rest. Site visits were conducted to identify potential study villages. Based on existing sociodemographic and LF prevalence data, 24 villages were identified and selected as study sites. Community sensitization meetings were conducted in each village to inform the community about the purpose, methodology, and significance of the proposed study and to obtain community consent. One day before the scheduled MDA and commencement of the study, communities were re-sensitized with the help of hamlet criers who used horn speakers or house-to-house visits to deliver information.

### 4.2. Study Design, Enrolment, and Sample Size

The study design was a prospective, longitudinal, active cohort event monitoring following the MDA of IA. As per the WHO and Tanzanian NTD program guidelines, any person aged ≥ 5 years living in an LF endemic region is eligible to receive MDA for the control and elimination of LF [1,2]. Pregnant women and children below five years of age were excluded from the study, as IA preventive chemotherapy is contraindicated in these groups. A cohort size of 10,000 individuals eligible to receive IA as MDA were enrolled. This sample size was determined based on the assumption that a cohort of 3000 individuals gives a 95% probability of identifying a single adverse event with an incidence of 1:1000 [13]. For a meaningful assessment, at-least three events need to be identified, hence the objective of obtaining a larger sample of 10,000 individuals [14,42].

### 4.3. Treatment and Safety Follow Up

On MDA Day, community drug distributors (CDDs) were accompanied by trained research assistants who administered pre-MDA questionnaires to consenting individuals and collected clinical data before drug intake. Baseline sociodemographic, clinical, and medical history, including any comorbidities, concomitant medications, and current clinical symptoms (Pre-MDA events), were collected and recorded before receiving the IA. Ivermectin (Merck Sharpe and Dohme, Haarlem, Netherlands), and albendazole (GlaxoSmithKline, Brentford, UK) tablets were from the national NTD programme (NTDCP). On the MDA day, study participants received a standard dose of Ivermectin based on height (roughly corresponding to 150–200 μg/kg) and albendazole 400 mg as recommended by the WHO [5]. MDA was conducted following routine national NTD programme procedures by using CDDs who delivered the medications using the directly observed treatment (DOT) approach at the households. The MDA campaign was led by NTDCP, and the study team had no role in the MDA planning, providing, or administering of the drugs. Study participants were actively followed up for any treatment-associated AEs through house-to-house visits on day one, two and seven following MDA.

### 4.4. Assessment and Severity Grading of Adverse Events

The primary study outcome was the incidence of any MDA-associated AEs (post-MDA AEs), defined as any outward medical event (sign, symptom, or disease) that occurred after drug intake and if the same type of event was not reported before drug intake (pre-MDA). In addition, an event reported on day two–day seven was also considered a valid AE if a participant experienced that event on any of the follow-up days but did not experience the same symptom on pre-MDA and preceding days. The secondary outcomes were the type and severity grade of AEs. Common Terminology Criteria for Adverse Events (CTCAE) version 5.0 [43] were used to grade the severity of observed AEs (1 to 5) as mild, moderate, severe, potentially life-threatening, and death as follows:

1. Grade 1: Mild; asymptomatic or mild symptoms; clinical or diagnostic observations only; intervention not indicated.
2. Grade 2: Moderate; minimal, local, or non-invasive intervention indicated; limiting age-appropriate Instrumental Activities of Daily Living (ADL).
3. Grade 3: Severe or medically significant but not immediately life-threatening; hospitalization or prolongation of hospitalization indicated; disabling; limiting self-care ADL.
4. Grade 4: Life-threatening consequences; urgent intervention indicated.
5. Grade 5: Death related to AE.

AEs recording was done through scheduled house to house visit during the follow-up period. The research team notified the supervising medical officer about any grade >2 AEs for further evaluation and management.

### 4.5. Data Management and Statistical Analysis

Data were collected electronically using tablets and uploaded on the central server located at the National Institute for Medical Research (NIMR) offices in Tanga. Data were initially collected on paper and later entered into the database using tablets. The Open-source Data Kit (ODK, https://opendatakit.org/ accessed on 12 March 2019) software was used to create the database and data collection applications. The data manager reviewed the collected data daily for completeness, and queries generated were sent back to the head of each study team for resolution.

Data were analyzed using STATA version 13.0 statistical software for analysis. Categorical variables were summarized as proportions, while continuous variables were summarized as mean with standard deviation (SD) or median and inter-quartile range. Categorical variables were compared using χ^2^-square tests. Both univariate and multivariate logistic regression models were used to determine factors associated with any AEs. The significance level was set at 0.05 and the confidence interval at 95%.

## 5. Conclusions

IA combination preventive chemotherapy is generally safe and tolerable. Treatment-associated AEs are mild-to-moderate and transient, resolving within a week of MDA. Although rare, Grade 3 severe drowsiness (0.9%) and dizziness (1.0%) in our study highlight the need to integrate pharmacovigilance in MDA campaigns for the timely detection and management of AEs. Being female, having pre-MDA clinical symptoms, chronic illnesses, in particular hypertension or asthma, and chronic manifestation of LF are significant risk factors for AEs following MDA of IA combination therapy. We recommend the safety follow-up of individuals with underlying clinical conditions and the integration of pharmacovigilance in MDA campaigns.

## Figures and Tables

**Table 1:** Baseline sociodemographic and clinical characteristics of study participants.

Variables (*N* = 9288)	Statistics
Age (years) median (Interquartile range)	26.0 (13.0–45.6)
Female sex, *n* (%)	4816 (51.9)
Body weight (kg), mean (SD) *	50.93 (19.63)
Height (cm), mean (SD)	149.24 (18.31)
Swollen arm, *n* (%)	52 (0.56)
Swollen leg, *n* (%)	122 (1.13)
Swollen breast (Males and Females), *n* (%)	18 (0.19)
Swollen scrotum (Males) *n* (%) **	148 (3.31)
Testicles/scrotum pain (Males), *n* (%)	119 (2.66)
Chronic manifestation of LF *n* (%)	389 (4.19)
Joint or muscle pain, *n* (%)	428 (4.61)
General body weakness, *n* (%)	216 (2.33)
Swelling/pain of armpit/groin, *n* (%)	61 (0.66)
Skin itching, *n* (%)	246 (2.65)
Skin rash, *n* (%)	102 (1.10)
Chronic illness	831 (8.95)
Use of bed nets	7804 (84.0)
Use of traditional medicines	139 (1.50)

* for weight = 1501, ** *n* for pain of testicles or scrotum = 4472.

**Table 2:** Severity grading of adverse events following ivermectin and albendazole MDA.

Adverse Event	Total Number of Events	Severity Grading		
		Grade 1 (Mild)	Grade 2 (Moderate)	Grade3 (Severe)
Headache	114	91 (79.8)	23 (20.2)	
Drowsiness	107	93 (86.9)	13 (12.1)	1 (0.9)
Fever	104	84 (80.8)	20 (19.2)	
Dizziness	98	86 (87.8)	11 (11.2)	1 (1.0)
Stomach pain	82	61 (74.4)	21 (25.6)	
Nausea	62	59 (95.2)	3 (4.8)	
Loss of appetite	45	42 (93.3)	3 (6.7)	
Diarrhea	43	33 (76.7)	10 (23.3)	
Coughing	32	30 (93.7)	2 (6.3)	
Breathing difficulty	14	10 (71.4)	4 (28.6)	
Vomiting	11	8 (72.7)	3 (27.3)	
Confusion	6	5 (83.3)	1 (16.7)	
Total	718	602 (83.8)	114 (15.9)	2 (0.3)

**Table 3:** Incidence and association of adverse events following mass administration of single dose Ivermectin and albendazole combinations.

Variable		Any MDA Associated Adverse Event (%)		χ^2^	*p*-Value
		No	Yes		
Sex	Male	4305 (96.3)	167 (3.7)	19.97	<0.001
	Female	4541 (94.3)	275 (5.7)		
Age group (years)	5–9	1913 (95.51)	90 (4.49)	1.73	0.63
	10–17	1413 (95.73)	63 (4.27)		
	18–64	4752 (95)	250 (5)		
	65+	752 (95.19)	38 (4.81)		
Used bed net	No	1680 (94.9)	90 (5.1)	0.52	0.47
	Yes	7153 (95.3)	351 (4.7)		
Used MDA last round	No	4341(95.4)	209 (4.6)	0.66	0.42
	Yes	4235(95.0)	221(5.0)		
Use of traditional medicines	No	8703 (95.2)	439 (4.8)	2.51	0.11
	Yes	132 (98.5)	2 (1.5)		
Chronic illness	No	8105 (95.0)	382 (4.5)	14.03	<0.001
	Yes	716 (92.5)	58 (7.5)		
Chronic LF manifestation	No	8502 (95.4)	413 (4.6)	7.80	0.005
	Yes	344 (92.2)	29 (7.8)		
Number of IA tablets taken	One	827 (95.4)	40 (4.6)	2.50	0.48
	Two	1397 (95.6)	64 (4.4)		
	Three	4336 (94.8)	238 (5.2)		
	Four	1941 (95.5)	92 (4.5)		
Ever used IA	Yes	4725 (95.3)	234 (4.7)	0.339	0.56
	No	2401(95.0)	127(5.0)		

**Table 4:** Predictors of adverse events following Ivermectin and albendazole combination preventive chemotherapy.

Variable		Univariate		Multivariate	
		OR (95%CI)	*p*-Value	OR (95%CI)	*p*-Value
Age group	18–64	1			
	59	0.85 (0.64–1.12)	0.25		
	10–17	0.89 (0.70–1.14)	0.38		
	18–64	0.96 (0.68–1.36)	0.821		
Sex	Male	1			
	Female	1.56 (1.28–1.90)	<0.001	1.55 (1.27–1.89)	<0.001
Used bed net	Yes	1			
	No	1.09 (0.86–1.39)	0.47		
Used MDA last round	Yes	1			
	No	0.92 (0.76–1.12)	0.42		
Used traditional medicines	No	1			
	Yes	0.300 (0.07–1.22)	0.07	0.26 (0.06–1.06)	0.06
Chronic illness	No	1			
	Yes	1.72 (1.29–2.29)	<0.001	1.61 (1.20–2.16)	0.001
Chronic LF manifestation	No	1			
	Yes	1.74 (1.17–2.57)	0.006	1.76 (1.18–2.62)	0.005
Number of ivermectin tablets taken	One	1			
	Two	0.95 (0.63–1.42)	0.79		
	Three	1.13 (0.81–1.60)	0.47		
	Four	0.98 (0.67–1.43)	0.92		
Ever used IA	No	1			
	Yes	0.93 (0.75–1.16)	0.56		
Use IA last MDA distribution	No	1			
	Yes	1.09 (0.89–1.32)	0.42		

**Table 5:** Descriptive and multivariate analysis (logistic) showing the association between any AEs and chronic conditions following MDA.

Variable		Proportion, *n*/*N* (%)	OR	95%CI	*p*-Value
Hypertension	No	412/8974 (4.6)	1		
	Yes	28/287 (9.8)	2.11	(1.40–3.18)	<0.001
Asthma	No	430/9159 (4.7)	1		
	Yes	10/102 (9.8)	1.98	(1.02–3.86)	0.045
Kidney problems	No	438/9239 (4.7)	1		
	Yes	2/22 (9.1)	1.30	(0.29–5.76)	0.73
Diabetes	No	439/9238 (4.7)	1		
	Yes	1/23 (4.3)	0.84	(0.11–6.31)	0.87
Tuberculosis	No	439/9240 (4.7)	1		
	Yes	1/21 (4.8)	1.04	(0.14–7.78)	0.97
Other chronic condition *	No	413/8943 (4.6)	1		
	Yes	29/345 (8.4)	1.78	(1.19–2.66)	0.005

* Ulcers (10.5%), hernia (5.5%), hypotension (5.5%), eye problems (3.9%), abdominal discomfort (3%), epilepsy (3%), chest pain (2.8%) and pelvic pain (1.9%).

## Data Availability

Data is contained within the article.
